# Impact of metabolic syndrome on the long-term prognosis of patients with hepatitis B virus-related hepatocellular carcinoma after hepatectomy

**DOI:** 10.3389/fonc.2022.1042869

**Published:** 2022-10-20

**Authors:** Kang-Jun Zhang, Tai-Wei Ye, Wen-Feng Lu, Fei-Qi Xu, Ya-Ming Xie, Dong-Dong Wang, Zun-Qiang Xiao, Si-Yu Liu, Wei-Feng Yao, Jian Cheng, Guo-Liang Shen, Jun-Wei Liu, Cheng-Wu Zhang, Dong-Sheng Huang, Lei Liang

**Affiliations:** ^1^ General Surgery, Department of Hepatobiliary and Pancreatic Surgery and Minimal Invasive Surgery, Zhejiang Provincial People’s Hospital, Affiliated People’s Hospital, Hangzhou Medical College, Hangzhou, China; ^2^ The Second School of Clinical Medicine, Zhejiang Chinese Medical University, Hangzhou, China; ^3^ Department of Hepatobiliary Surgery, Eastern Hepatobiliary Surgery Hospital, Second Military Medical University (Navy Medical University), Shanghai, China; ^4^ Department of Medical, Lishui Municipal Central Hospital, Lishui, Zhejiang, China; ^5^ Key Laboratory of Tumor Molecular Diagnosis and Individualized Medicine of Zhejiang Province, Zhejiang Provincial People's Hospital, Hangzhou, Zhejiang, China; ^6^ Department of Clinical Medicine, Hangzhou Medical College, Hangzhou, Zhejiang, China

**Keywords:** metabolic syndrome, hepatitis virus B, hepatocellular carcinoma, hepatectomy, survival

## Abstract

**Background & aims:**

The long-term prognosis of patients with metabolic syndrome (MS) and hepatitis B virus-related hepatocellular carcinoma (HBV-HCC) after radical hepatectomy remains unclear. The purpose of this study was to elucidate the effect of MS on long-term survival for patients with HBV-related HCC after hepatectomy.

**Methods:**

Patients with HBV-HCC after hepatectomy were included. Patients were stratified into MS-HBV-HCC and HBV-HCC groups. Clinical features and surgical outcomes were compared between the two groups, and COX regression analysis was used to determine independent risk factors associated with overall survival (OS) and recurrence-free survival (RFS).

**Result:**

389 patients (MS-HBV-HCC group: n=50, HBV-HCC group: n=339) were enrolled for further analysis. Baseline characteristics showed that patients with MS-HBV-HCC were associated with a high rate of elderly patients, ASA score, and co-morbid illness, but a lower rate of anatomy hepatectomy. There were no significant differences in perioperative complications. After excluding patients who relapsed or died within 90 days after surgery, multivariate Cox regression analysis showed MS was an independent risk factor of OS (HR 1.68, 95% CI 1.05-2.70, P = 0.032) and RFS (HR 1.78, 95% CI 1.24-2.57, P = 0.002).

**Conclusion:**

MS is an independent risk factor for poor OS and RFS in HBV-infected HCC patients after radical hepatectomy. This suggests that we need to strengthen postoperative follow-up of the relevant population and encourage patients to develop a healthy lifestyle.

## Introduction

Primary liver cancer remains the sixth most common cancer worldwide and is one of the top three causes of cancer-related deaths, with hepatocellular carcinoma (HCC) accounting for 75-85% (1, 2). Hepatitis B virus (HBV) infection is the most important risk factor for HCC development in the Asia-Pacific region, especially in China. Current guidelines have recommended routine antiviral therapy for HCC patients with HBV infection after surgery (3), but the recurrence rate is still high. Therefore, it is necessary to actively explore the risk factors affecting long-term survival after surgery.

Metabolic syndrome (MS) is a clinical syndrome of obesity, dyslipidemia (low high-density lipoproteinemia and/or high triglyceridemia), hyperglycemia (diabetes or impaired glucose regulation), and hypertension (4, 5). Previous studies have shown that hepatectomy for MS-HCC has a higher but acceptable operative risk, but the long-term survival outcome remains controversial (6–11). Reasons for the controversy include a small sample size or an uneven baseline of enrolled patients. Studies focusing on one factor in MS may also lead to biased results because many factors are related and should be analyzed comprehensively (12–14). Furthermore, patients with early recurrence and death, especially within 90 days, mainly due to surgical and tumor factors (15), should be excluded. Most HCC patients in the Asia-Pacific region have a background of chronic HBV infection, and the long-term prognosis of HBV-HCC patients with MS is still unclear.

The aim of this study is to elucidate the effect of MS on long-term survival after radical hepatectomy in HBV-infected HCC patients, excluding patients who relapsed or died within 90 days after surgery.

## Material and methods

### Study population

Data from a Chinese large single-center database of patients who underwent curative-intent liver resection for HBV-infected HCC between January 2011 and January 2018 at Zhejiang Provincial People’s Hospital. MS is diagnosed when at least three of the following conditions are met: abdominal obesity (waist circumference ≥ 90cm in men; ≥ 80 cm for women); dyslipidemia (triglyceride ≥ 1.70 mmol/L or high-density lipoprotein cholesterol < 1.04 mmol/L); diagnosed with diabetes or glucose intolerance (fasting blood glucose ≥ 6.1 mmol/L); treated for hypertension or hypertension (blood pressure ≥ 130/≥ 85 mmHg); abdominal obesity with a body mass index (BMI) ≥ 25.0 kg/m^2^ (16). Patients younger than 18 years of age, with recurrent HCC, concomitant tumors, incomplete clinical data, concomitant HCV or no concomitant HBV infection were excluded. All patients underwent R0 resection, in which all microscopic and gross tumors were removed.

Selection criteria for hepatectomy for HCC were constant during the study period, such as previously reported tumor location and number, liver function reserve, and future residual liver volume (17, 18). This study was approved by the Institutional Evaluation Committee of Zhejiang Provincial People’s Hospital according to the Helsinki Declaration (No. QT2022238).

### Baseline characteristics and follow-up

There were seven patient-related variables: age, sex, smoking history, Performance status (PS), American Society of Anesthesiologists (ASA) score, co-morbid illness, and preoperative anti-HBV therapy. There were seven liver-related and laboratory variables: preoperative HBV-DNA levels, alanine aminotransferase (ALT), aspartate aminotransferase (AST), alpha-fetoprotein (AFP), cirrhosis, portal hypertension, and Child-Pugh grade. There were six tumor-related and pathological variables: maximum tumor diameter, tumor number, satellite nodules, vascular invasion (macro- or micro-), tumor differentiation (good/moderate or poor), and tumor encapsulation (incomplete or complete). There were five surgery-related variables: intraoperative blood loss, intraoperative blood transfusion, the scope of liver resection (minor or major), type of liver resection (anatomic or non-anatomic) (19), and resection margin.

After hepatectomy, patients were followed up according to a standardized recurrence surveillance protocol. The date of tumor recurrence, date of death and cause of death, and date of last follow-up were recorded. Short-term outcomes included postoperative hospital stay, postoperative morbidity, severe morbidity (20), postoperative 90-day mortality, and postoperative 90-day death or recurrence.

### Statistical analysis

SPSS 25.0 software was used for statistical analysis of the data. Continuous variables were represented by mean or median (range), and categorical variables were represented by numbers (n, %). Continuous variables were compared using Student’s T test or Mann-Whitney U test and comparisons between categorical variables were performed using either χ2 test or Fisher’s exact test, as appropriate. OS and RFS were calculated using the Kaplan-Meier method, and multivariate COX regression was used to determine whether MS was an independent risk factor for worse OS and RFS. P < 0.05 was considered statistically significant.

## Results

### Baseline characteristics

Of the 559 patients treated with hepatectomy for HCC at our center, 170 patients did not meet the inclusion criteria and were excluded. 389 HBV-infected HCC patients were collected for further analysis. Among them, 339 (87.1%) patients without MS and 50 (12.9%) patients with MS. **Table 1** shows that the proportion of patients with MS complicated with anatomic liver resection was lower (32.0% vs. 49.3%, P = 0.022), but the proportion of patients with age ≥ 60 years, ASA score > 2 and co-morbid illness was higher (both P < 0.05). There were no significant differences in postoperative hospital stay, postoperative 30-day morbidity, serious complications, postoperative 90-day mortality, and postoperative 90-day death or recurrence between the two groups (all P > 0.05).

**Table 1:** Comparison of clinical and pathological characteristics and short-term outcomes between the study groups in the whole study population.

Variables (N, %)	MS- HBV-HCCN = 50	HBV-HCCN = 339	P
Male Sex	46 (92.0)	294 (86.7)	0.294
Age ≥ 60 years	26 (52.0)	121 (35.7)	**0.026**
Smoking history	23 (46.0)	140 (41.3)	0.529
PS score ≥ 1	10 (20.0)	77 (22.7)	0.667
ASA score > 2	15 (30.0)	61 (18.0)	**0.046**
Co-morbid illness^a^	20 (40.0)	29 (8.6)	**< 0.001**
Child-Pugh grade B	4 (8.0)	35 (10.3)	0.609
Preoperative HBV-DNA >10^4^ copies/ml	12 (24.0)	100 (29.5)	0.423
Preoperative anti-HBV therapy	13 (26.0)	82 (24.2)	0.781
Preoperative ALT > 40 U/L	21 (42.0)	113 (33.3)	0.229
Preoperative AST > 40 U/L	22 (44.0)	142 (41.9)	0.778
Preoperative AFP > 400 ug/L	12 (24.0)	95 (28.0)	0.552
Cirrhosis	41 (82.0)	282 (83.2)	0.835
Portal hypertension	9 (18.0)	93 (27.4)	0.157
Maximum tumor size > 5cm	21 (42.0)	115 (33.9)	0.264
Multiple tumors	13 (26.0)	66 (19.5)	0.284
Macroscopic vascular invasion	4 (8.0)	24 (7.1)	0.814
Microscopic vascular invasion	23 (46.0)	163 (48.1)	0.783
Satellite nodules	5 (10.0)	43 (12.7)	0.590
Poor tumor differentiation	38 (76.0)	268 (79.1)	0.622
None or incomplete tumor encapsulation	32 (64.0)	199 (58.7)	0.476
Intraoperative blood loss > 400 ml	15 (30.0)	127 (37.5)	0.306
Intraoperative blood transfusion	12 (24.0)	111 (32.7)	0.215
Anatomical resection	16 (32.0)	167 (49.3)	**0.022**
Major hepatectomy	15 (30.0)	95 (28.0)	0.772
Resection margin < 1 cm	10 (20.0)	60 (17.7)	0.693
Postoperative hospital stay (day)^b^	9 (7-12)	9 (7-12)	0.936
Postoperative 30-day morbidity	27 (54.0)	169 (49.9)	0.584
Postoperative severe morbidity	4 (8.0)	33 (9.7)	0.696
Postoperative 90-day mortality	2 (4.0)	5 (1.5)	0.210
Postoperative 90-day death or recurrence	2 (4.0)	20 (5.9)	0.587

PS, Performance status; ASA, American Society of Anesthesiologists; HBV, Hepatitis B virus; ALT, Alanine aminotransferase; AST, Aspartate aminotransferase; AFP, Alpha-fetoprotein; ^a^Co-morbid illnesses include hypertension, diabetes mellitus, cardiovascular disease, chronic obstructive pulmonary disease, and renal dysfunction.^b^Continuous variables are expressed as medians and interquartile ranges. The bold values mean that those variables are statistically significant.

### Overall survival and recurrence-free survival

**Figure 1:**
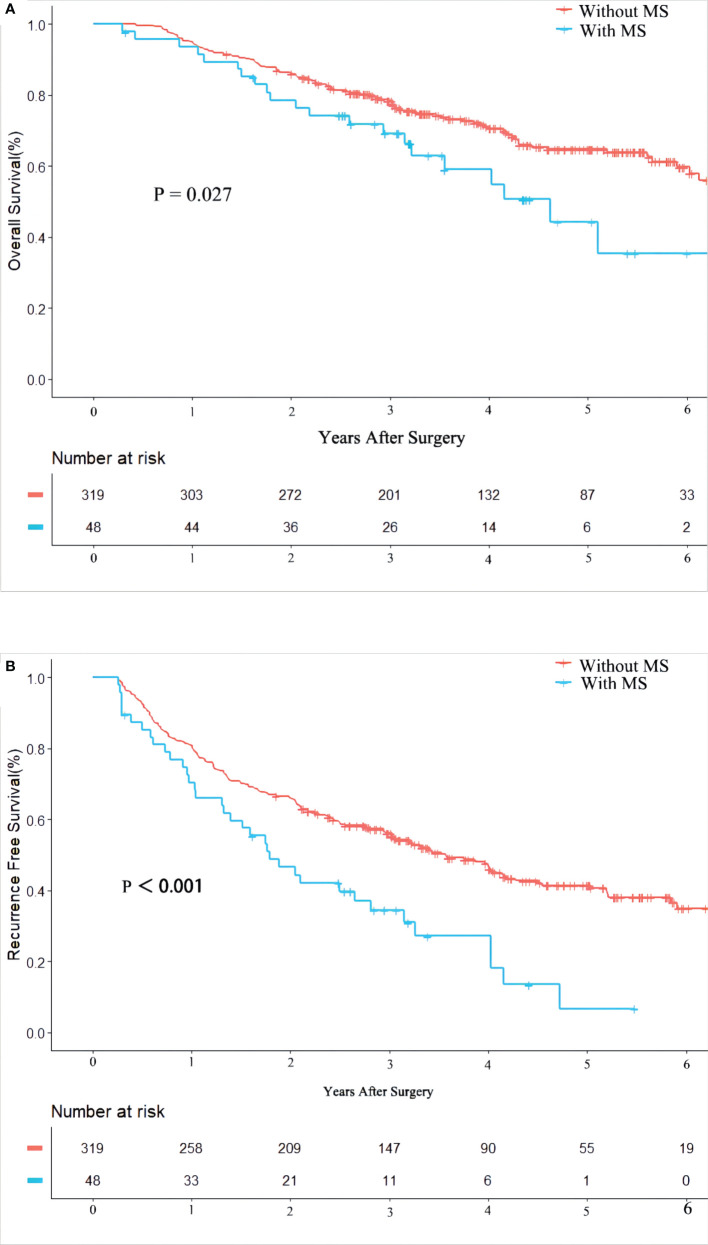
Kaplan-Meier Curves of overall survival **(A)** and recurrence free survival **(B)** between HBV-infected patients with and without metabolic syndrome.

Patients who died or relapsed within 90 days were excluded from our study cohort to reduce the impact of tumor and surgery (n = 22). At a median follow-up of 40.2 months, 123 (33%) and 196 (53%) patients died and HCC recurred, respectively. **Table 2** shows that the 1-, 3- and 5-year OS of patients with or without MS after radical hepatectomy for HCC were 91.7%, 54.2% and 12.5%, and 95.0%, 63.0% and 27.3%, respectively. RFS of 1-, 3- and 5-year were 68.8%, 22.9% and 2.1%, and 80.9%, 46.1% and 17.2%, respectively. Patients with MS had poor 5-year OS, 3-year RFS, and 5-year RFS (all P < 0.05). In **Figure 1**, Kaplan-Meier method was used to compare OS and RFS curves of the two groups. By log-rank test, OS and RFS of HCC patients with MS were worse than those without MS (hazard ratio (HR) 1.69, 95% confidence interval (CI) 1.06 – 2.71, P = 0.027; HR 1.92, 95% CI 1.34 – 2.75, P < 0.001, respectively).

**Table 2:** Comparisons of long-term outcomes between HBV-infected patients with and without metabolic syndrome.

Variables (N, %)	MS & HBV-HCCN = 48	HBV-HCCN = 319	P*
Death during the follow-up	21 (43.8)	102 (32.0)	0.107
Recurrence during the follow-up	30 (62.5)	166 (52.0)	0.176
OS			
1-year OS rate	44 (91.7)	303 (95.0)	0.345
3-year OS rate	26 (54.2)	201 (63.0)	0.240
5-year OS rate	6 (12.5)	87 (27.3)	0.028
RFS			
1-year RFS rate	33 (68.8)	258 (80.9)	0.053
3-year RFS rate	11 (22.9)	147 (46.1)	0.003
5-year RFS rate	1 (2.1)	55 (17.2)	0.006

*Death or recurrence within 90 days after surgery was excluded. OS, overall survival; RFS, recurrence-free survival.

### Univariable and multivariable analyses

**Table 3** and **Table 4** showed the univariate and multivariate COX-regression analysis results of OS and RFS in patients with HBV-infected HCC after hepatectomy. The results showed that MS was an independent risk factor for worse OS and RFS (HR 1.68, 95% CI 1.05 – 2.70, P = 0.032; HR 1.78, 95% CI 1.24 – 2.57, P = 0.002). Furthermore, the results also showed PS score ≥ 1, preoperative HBV-DNA level > 10^4^ copies/ml, macroscopic vascular invasion, microscopic vascular invasion, satellite nodules, incomplete tumor encapsulation, and resection margin < 1cm were independent risk factors associated with poorer OS. Meanwhile, preoperative HBV-DNA level > 10^4^ copies/ml, tumor size, multiple tumors, macroscopic vascular invasion, microscopic vascular invasion, satellite nodules, incomplete tumor encapsulation, and resection margin < 1cm were independent risk factors associated with worse RFS (all P < 0.05).

**Table 3:** Univariable and multivariable Cox regression analyses on risk factors of overall survival.

Variables	HR Comparison	UV HR (95% CI)	UV P	MV HR (95% CI)	MV P*
Metabolic syndrome	Yes vs. No	1.69 (1.06 - 2.71)	0.027	1.68 (1.05 - 2.70)	0.032
Sex	Male vs. Female	1.12 (0.64 - 1.97)	0.684		
Age	**≥**60 vs. < 60years	1.03 (0.71 - 1.48)	0.896		
Smoking history	Yes vs. No	1.00 (0.70 - 1.43)	0.996		
PS score	≥ 1 vs. 0	2.90 (2.01 - 4.17)	<0.001	1.64 (1.09 - 2.45)	0.017
ASA score	> 2 vs. ≤ 2	1.31 (0.86 - 2.00)	0.208		
Co-morbid illness	Yes vs. No	1.44 (0.86 - 2.40)	0.165		
Child-Pugh grade	B vs. A	1.51 (0.91 - 2.49)	0.109		
HBV-DNA level	>10^4^ vs. ≤10^4^ copies/ml	2.09 (1.46 - 2.99)	<0.001	2.00 (1.39 - 2.87)	<0.001
Anti-HBV therapy	Yes vs. No	0.58 (0.36 - 0.94)	0.028	NS	
Preoperative ALT level	> 40 vs. ≤ 40 U/L	1.69 (1.18 - 2.41)	0.004	NS	
Preoperative AST level	> 40 vs. ≤ 40 U/L	1.83 (1.29 - 2.61)	0.001	NS	
Preoperative AFP level	> 400 vs. ≤ 400 ug/L	1.48 (1.02 - 2.15)	0.041	NS	
Cirrhosis	Yes vs. No	0.93 (0.59 - 1.46)	0.735		
Portal hypertension	Yes vs. No	1.06 (0.72 - 1.56)	0.779		
Maximum tumor size	> 5 vs. ≤ 5cm	2.13 (1.50 - 3.04)	<0.001	NS	
Multiple tumors	Yes vs. No	2.11 (1.43 - 3.11)	<0.001	NS	
Macroscopic vascular invasion	Yes vs. No	3.20 (1.79 - 5.71)	<0.001	1.79 (1.31 - 2.92)	0.021
Microscopic vascular invasion	Yes vs. No	2.89 (2.00 - 4.18)	<0.001	1.81 (1.18 - 2.77)	0.006
Satellite nodules	Yes vs. No	2.51 (1.58 - 4.00)	<0.001	1.71 (1.15 - 2.53)	0.008
Tumor differentiation	Poor vs. Well/moderately	1.74 (1.15 -2.63)	0.009	NS	
Tumor encapsulation	Incomplete vs. Complete	2.98 (2.00 - 4.46)	<0.001	1.94 (1.25 - 3.03)	0.003
Intraoperative blood loss	> 400 vs. ≤ 400ml	2.27 (1.59 - 3.23)	<0.001	NS	
Blood transfusion	Yes vs. No	2.10 (1.47 - 2.99)	<0.001	NS	
Anatomical resection	Yes vs. No	1.23 (0.86 - 1.76)	0.249		
Major hepatectomy	Major vs. Minor	2.20 (1.53 - 3.17)	<0.001	NS	
Resection margin	<1 vs. ≥1cm	2.14 (1.44 - 3.18)	<0.001	2.46 (1.63 - 3.71)	<0.001

*Death or recurrence within 90 days after surgery was excluded. PS, Performance status; ASA, American Society of Anesthesiologists; HBV, Hepatitis B virus; ALT, Alanine aminotransferase; AST, Aspartate aminotransferase; AFP, Alpha-fetoprotein; HR, hazard ratio; CI, confidence interval; MV, multivariable; UV, univariable; NS, no significance.

**Table 4:** Univariable and multivariable Cox regression analyses on risk factors of recurrence-free survival.

Variables	HR Comparison	UV HR (95% CI)	UV P	MV HR (95% CI)	MV P*
Metabolic syndrome	Yes vs. No	1.92 (1.34 - 2.75)	<0.001	1.78 (1.24 - 2.57)	0.002
Sex	Male vs. Female	1.26 (0.80 - 1.98)	0.318		
Age	**≥**60 vs. < 60years	1.05 (0.80 - 1.39)	0.717		
Smoking history	Yes vs. No	1.16 (0.88 - 1.52)	0.297		
PS score	≥ 1 vs. 0	2.34 (1.74 - 3.15)	<0.001	NS	
ASA score	> 2 vs. ≤ 2	1.22 (0.87 - 1.69)	0.247		
Co-morbid illness	Yes vs. No	1.47 (0.99 - 2.19)	0.058		
Child-Pugh grade	B vs. A	1.39 (0.93 - 2.08)	0.113		
HBV-DNA level	>10^4^ vs. ≤10^4^ copies/ml	1.89 (1.43 - 2.50)	<0.001	1.62 (1.19 - 2.21)	0.002
Anti-HBV therapy	Yes vs. No	0.75 (0.53 - 1.04)	0.086		
Preoperative ALT level	> 40 vs. ≤ 40 U/L	1.85 (1.41 - 2.44)	<0.001	NS	
Preoperative AST level	> 40 vs. ≤ 40 U/L	2.13 (1.62 - 2.80)	<0.001	1.47 (1.09 - 2.00)	0.012
Preoperative AFP level	> 400 vs. ≤ 400 ug/L	1.27 (0.94 - 1.71)	0.117		
Cirrhosis	Yes vs. No	1.023 (0.72 - 1.45)	0.899		
Portal hypertension	Yes vs. No	1.06 (0.79 - 1.43)	0.693		
Maximum tumor size	> 5 vs. ≤ 5cm	1.91 (1.46 - 2.52)	<0.001	1.45 (1.07 - 1.96)	0.015
Multiple tumors	Yes vs. No	2.36 (1.73 - 3.22)	<0.001	1.70 (1.22 - 2.36)	0.002
Macroscopic vascular invasion	Yes vs. No	2.87 (1.76 - 4.65)	<0.001	1.87 (1.36 - 2.51)	0.014
Microscopic vascular invasion	Yes vs. No	2.46 (1.86 - 3.25)	<0.001	1.47 (1.06 - 2.04)	0.021
Satellite nodules	Yes vs. No	2.38 (1.63 - 3.48)	<0.001	1.41 (1.02 - 1.95)	0.038
Tumor differentiation	Poor vs. Well/moderately	1.71 (1.24 - 2.37)	0.001	NS	
Tumor encapsulation	Incomplete vs. Complete	2.65 (1.97 - 3.56)	<0.001	2.01 (1.43 - 2.82)	<0.001
Intraoperative blood loss	> 400 vs. ≤ 400ml	1.80 (1.37 - 2.37)	<0.001	NS	
Blood transfusion	Yes vs. No	2.09 (1.59 - 2.75)	<0.001		
Anatomical resection	Yes vs. No	1.22 (0.93 - 1.60)	0.150		
Major hepatectomy	Major vs. Minor	2.22 (1.66 - 2.95)	<0.001	NS	
Resection margin	<1 vs. ≥1cm	1.88 (1.36 - 2.58)	<0.001	1.84 (1.33 -2.55)	<0.001

*Death or recurrence within 90 days after surgery was excluded. PS, Performance status; ASA, American Society of Anesthesiologists; HBV, Hepatitis B virus; ALT, Alanine aminotransferase; AST, Aspartate aminotransferase; AFP, Alpha-fetoprotein; HR, hazard ratio; CI, confidence interval; MV, multivariable; UV, univariable; NS, no significance.

## Discussion

In this study, a total of 389 patients with HCC complicated with HBV infection were enrolled to evaluate the effect of MS on long-term survival after radical hepatectomy. According the excluding criterion, 389 patients were finally included for further analysis. HBV-infected HCC patients with MS were mostly overweight elderly males with solitary large tumors (92.0% male, 52.0% ≥ 60 years old, 74.0% single tumor and 42.0% > 50 mm). In the multivariate COX regression analysis, MS was an independent risk factor affecting OS (HR 1.68, 95% CI 1.05–2.70, P = 0.032) and RFS (HR 1.78, 95% CI 1.24–2.57, P = 0.002). In other words, MS increased nearly 1.7 folds risk of tumor recurrence and death.

At present, hepatectomy is still the preferred method for radical treatment of HCC. However, the postoperative recurrence rate was still high, with an overall recurrence rate of more than 70% within 5 years (17, 21, 22). At the same time, studies have shown that the causes of patients’ early recurrence and death, especially within 90 days, are mainly surgical and tumor factors (15). Although the development of neoadjuvant and adjuvant therapies has improved the prognosis of liver cancer, there is no definite treatment plan. Therefore, actively exploring the risk factors of long-term recurrence and death after hepatectomy is an important clinical topic to be solved urgently.

MS is associated with a variety of tumors and has a long-term impact on patient survival. Because NAFLD is often associated with insulin resistance, central obesity, dyslipidemia, hypertension, and hyperglycemia, it is often considered a hepatic manifestation of the MS (23–26). A growing body of evidence suggests that the relationship between NAFLD and MS is bidirectional, exacerbating each other’s conditions (27). NAFLD progresses to non-alcoholic steatohepatitis (NASH) due to hepatic steatosis and chronic substrate overload leading to lipotoxicity (28). Hepatic lipid accumulation leads to cellular metabolic changes and accumulation of potentially toxic metabolites leading to the occurrence of liver tumors (29–31).

Similar to the characteristics of metabolism-related HCC previously reported (6, 7, 11, 32), HBV-infected HCC patients with MS were more likely to be overweight elderly men with a single large tumor (92.0% male, 52.0% ≥ 60 years old, 74.0% single tumor and 42.0% > 50 mm). Comorbidity, older age, and high-risk lifestyle behaviors [such as sedentary behavior or unbalanced diet (33)] associated with MS further hinder tumor management.

MS-HCC can occur in non-cirrhotic liver parenchyma in previous studies (2, 6, 11), but there was no difference in cirrhosis between the two groups in this study (82.0% vs. 83.2%, P=0.835). This is related to the co-infection with HBV in this study population, and studies have shown that MS has a detrimental effect on the course of viral hepatitis, especially by accelerating the progression of liver fibrosis (34).

Previous studies have reported that hepatectomy for patients with MS is safe, feasible and beneficial to prognosis, although the probability of postoperative complications is higher (6, 8, 10, 35, 36). Similarly, the incidence of postoperative complications was also high among HBV-infected HCC patients with MS in our cohort, although there was no difference between the two groups (52.1% vs. 49.8%, P= 0.772). This may be due to the high proportion of patients with cirrhosis in both groups, even up to 80%, which is similar to the characteristics of HBV-HCC (7). A high proportion of cirrhosis is closely related to a high incidence of complications (37, 38).

The long-term prognosis of HBV-HCC patients with MS after radical hepatectomy remains unclear. In 2018, Tian Y et al. compared hepatectomy in MS-HCC patients with HBV-HCC patients. Patients were stratified according to the AJCC Cancer Staging Manual, Seventh Edition (2010) to compare the long-term outcomes of MS-HCC, MS-HBV-HCC, and HBV-HCC patients. It was found that compared with the other two groups, MS-HCC patients in AJCC stage I had higher RFS and OS, and there was no significant difference in RFS and OS in MS-HCC, MS-HBV-HCC and HBV-HCC patients in AJCC stage II, III and IV (7). In 2020, Tian Y et al. in the study of BCLC stage 0 or A HCC patients, compared with patients without MS, the long-term survival of most HBV-related HCC patients with hepatectomy in the presence of MS was comparable (10). In addition, a multi-center retrospective study by Viganò et al. found that the prognosis of the MS-HCC cohort was better than that of the HCV-HCC cohort after a propensity matching analysis (6). However, factors such as obesity and diabetes are often associated with a worse prognosis (12–14). The components of MS are increasingly linked to a variety of cancers, including increased disease risk and worsening outcomes. In this cohort, death and recurrence within 90 days after surgery were excluded, effectively controlling the direct impact of the tumor itself and surgery. We found that 5-year OS and RFS were significantly worse in HBV-HCC patients with MS (12.5% vs. 27.3%, P = 0.028; 2.1% vs. 17.2%, P = 0.006 respectively). By multivariate Cox regression analysis, MS was determined to be an independent risk factor for OS and RFS. In addition, preoperative HBV-DNA level > 10^4^ copies/ml, microscopic vascular invasion, tumor encapsulation (none or incomplete), resection margin < 1cm were also independent risk factors for worse OS and RFS in HBV-infected HCC patients (all P < 0.05).

The diagnosis of MS is cumbersome and often goes unnoticed. The gold standard for diagnosing NAFLD is pathological examination of liver tissue. Once cirrhosis has progressed, diagnosis becomes more difficult due to the loss of fat (39). At present, there is no specific diagnostic method, so the prevention of MS and the treatment after the occurrence of HCC are particularly critical. Abdominal ultrasound monitoring is recommended every 4-6 months in all patients with cirrhosis (40). Of course, if technology develops in the future, more sensitive and cheaper serum tests should be used for surveillance.

There are currently no approved drugs to treat MS/NAFLD, and diet, lifestyle changes, and exercise remain the mainstay of treatment (41). There is no doubt that hepatitis B virus infection must also receive regular antiviral treatment. In elderly patients with MS, cardiopulmonary function tests must be fully evaluated before surgery. Attention must be paid to perioperative management of these patients, and timely and effective treatment of postoperative complications is needed. Regular follow-up tests after surgery must also be emphasized.

There are some defects in this study. First of all, this study is retrospective and there are some unavoidable biases. Secondly, the cases in this study were all infected with HBV, so whether it can be applied to patients infected with HCV needs further study.

## Conclusion

In conclusion, this study demonstrates that MS is an independent risk factor for worse OS and RFS in HBV-infected patients after radical hepatectomy for HCC. This suggests that we need to strengthen postoperative follow-up of the relevant population and encourage patients to develop a healthy lifestyle.

## Data availability statement

The original contributions presented in the study are included in the article/supplementary material. Further inquiries can be directed to the corresponding authors.

## Author contributions

K-JZ, T-WY, W-FL, and F-QX contributed equally to this work. LL and D-SH had full access to all the data in the study and takes responsibility for the integrity of the data and the accuracy of the data analysis. Study concept and design: K-JZ, LL, and D-SH. Acquisition, analysis, or interpretation of data: T-WY, W-FL, F-QX, Y-MX, D-DW, Z-QX, S-YL, W-FY, JC, and G-LS. Drafting of the manuscript: K-JZ and T-WY. Critical revision of the manuscript for important intellectual content: J-WL and C-WZ. Statistical analysis: T-WY, D-DW and Y-MX. Obtained funding: LL. Administrative, technical, or material support: C-WZ, D-SH, and LL. Study supervision: D-SH, LL. All authors contributed to the article and approved the submitted version.

## Funding

This work was supported by Zhejiang Provincial People’s Hospital (No. ZRY2020A004), and the Health Commission of Zhejiang Province (No.2022KY532).

## Conflict of interest

The authors declare that the research was conducted in the absence of any commercial or financial relationships that could be construed as a potential conflict of interest.

The reviewer CL declared a shared affiliation with the author W-FL to the handling editor at the time of review.

## Publisher’s note

All claims expressed in this article are solely those of the authors and do not necessarily represent those of their affiliated organizations, or those of the publisher, the editors and the reviewers. Any product that may be evaluated in this article, or claim that may be made by its manufacturer, is not guaranteed or endorsed by the publisher.

## Abbreviations

MS, metabolic syndrome; HBV, hepatitis virus B; HCC, hepatocellular carcinoma; NAFLD, non-alcoholic fatty liver disease; NASH, non-alcoholic steatohepatitis; ASA, American Society of Anesthesiologists; OS, overall survival; RFS, recurrence-free survival; PS, performance status; ALT, alanine aminotransferase; AST, aspartate transaminase; AFP, alpha-fetoprotein; HR, hazard ratio; CI, confidence interval; MV, multivariable; UV, univariable.
